# Formulation Attributes Impact Immune Profile of an Oral and Thermostable COVID-19 Subunit Vaccine

**DOI:** 10.3390/vaccines12101087

**Published:** 2024-09-24

**Authors:** Elodie Burlet, Nissy Thomas, Shanna Carwell, Brett W. Gershman, Garry L. Morefield

**Affiliations:** VaxForm, LLC., Bethlehem, PA 18015, USA; elodie.burlet@vaxform.com (E.B.);

**Keywords:** COVID-19 vaccine, mucosal immunity, oral subunit vaccine, thermostable vaccine, powder vaccine

## Abstract

While approved vaccines for COVID-19 provide protection against severe disease and death, they have limited efficacy in the prevention of infection and virus transmission. Mucosal immunity is preferred over systemic immunity to provide protection at the point of entry against pathogens such as SARS-CoV-2. VaxForm has developed an oral vaccine delivery platform that elicits mucosal and systemic immune responses by targeting immune cells in the gut through C-type lectin receptors. The technology consists of microencapsulating the vaccine with an enteric polymer, which also results in enhanced thermostability. This article describes the formulation development and in vivo testing of a novel protein-based oral COVID-19 vaccine using this technology. Results demonstrate successful induction of immune response in mice and showed that the particle size of the vaccines following administration can impact the ratio of mucosal to systemic response. Immunogenicity and thermostability of liquid suspension and dry powder versions of the vaccine were compared in mice. The liquid suspension vaccine showed excellent heat resistance by maintaining immunogenicity after 14 days of storage at 60 °C. While further investigation is needed to determine correlates of protection and duration of response for mucosal immunity, this study demonstrates the vaccine’s potential as a COVID-19 booster to enhance mucosal protection in humans and improve global access by lowering the cost of production, removing cold-chain requirements, and allowing self-administration.

## 1. Introduction

As of July 2024, over 7 million people have died from COVID-19, and more than 775 million people have been infected globally [1]. While approved vaccines reduce the risk of severe disease and death, they offer suboptimal protection from infection, allowing the SARS-CoV-2 virus to continue to circulate and mutate. Additionally, these vaccines require aseptic manufacturing, cold or ultra-cold chain storage, and administration by a medical professional, all of which limit global access. Next-generation vaccines are needed to lower virus transmission rates and improve global coverage. It is well-established that inducing mucosal immunity, the first line of defense, is preferred over systemic immunity to combat mucosal pathogens [2]. Oral vaccine administration strategies can address these unmet needs by eliciting robust mucosal immunity and removing the need for needles and medical professionals to administer the vaccines [3]. Intranasal vaccines, such as the live-attenuated influenza vaccine FluMist^®^, while providing good efficacy, still need a device for administration, adding development and regulatory challenges. Furthermore, understanding of the nasal barriers is poor, and intranasal immunization has been associated with increased risk of Bells Palsy [4]. Despite oral immunization being seen as the ideal and safest method of vaccination, only a handful of oral vaccines are approved for human use. These vaccines target enteric pathogens and use older platforms such as live attenuated organisms and inactivated whole cells or viruses. Developing an oral vaccine with newer vaccine modalities such as protein-based or RNA-based vaccines is very challenging due to the hostile environment of the stomach. Despite these challenges, oral administration remains attractive from an immunological perspective as the gastrointestinal tract contains about 30 m^2^ of mucosal surface that is richly endowed with immune inductive tissue, such as Peyer’s patches [5]. Additionally, immunization at one mucosal site can result in antibody secretion systemically, as well as at other selected mucosal sites [2,6,7].

There is a need for new platforms that allow oral administration of modern vaccine modalities such as subunit proteins, DNA, and RNA. VaxForm has developed a vaccine delivery platform that allows oral administration of subunit protein vaccines and ambient temperature storage. The novel platform consists of adsorbing a C-type lectin receptor (CTL) agonist and the antigen of interest to aluminum adjuvant particles. The CTL agonist targets the vaccine particles to dendritic cells, macrophages, and microfold (M) cells in the oral cavity and the Peyer patches in the Gut Associated Lymphoid Tissue (GALT) [8,9]. The aluminum particles make the vaccine a suitable size for efficient internalization by these cells through phagocytosis [10]. Protection of the vaccine from degradation in the stomach is provided by encapsulating the vaccine particles with a pH-dependent enteric polymer that protects the vaccine. Microencapsulation of the vaccine with the polymer also imparts thermostability to the vaccine.

In this study, the goal was to apply the described oral delivery platform using the recombinant Receptor Binding Domain (RBD) subunit of the spike protein as an antigen to develop a highly stable, low-cost COVID-19 vaccine in comparison to the mRNA vaccines that were being developed at the time (summer 2020). This paper describes the formulation development of the vaccine candidate and the immunogenicity studies performed to compare the thermostability of liquid suspension versus dry powder presentation of the vaccine.

## 2. Materials and Methods

### 2.1. Recombinant Receptor Binding Domain (RBD) Production

The SARS-CoV-2 RBD (amino acids 331 to 524) sequence from the original Wuhan Strain was subcloned into the pET-24a(+) plasmid by GenScript, Inc. with Isopropyl β-D-1-thiogalactopyranoside (IPTG) inducible expression and kanamycin resistance as a bacterial selection marker. The plasmid was transformed into a competent *Escherichia coli* (*E. coli*) strain (BL21 DE3) (New England Biolabs, Ipswich, MA, USA, #C35271) using the manufacturer’s heat-shock-mediated transformation protocol. *E. coli* cells containing the RBD expression plasmid were then selected to produce a research cell bank. To produce the recombinant RBD, transformed *E. coli* were grown in 30 g/L animal-free Soytone broth (Teknova, Hollisters, CA, USA, catalog # S9052) with 5 g/L yeast extract (VWR, catalog #J850) and 50 µg/mL kanamycin (Tokyo Chemical Industry, Tokyo, Japan, catalog #K0047) at 37 °C until OD600 reaches 0.5–0.9 (log phase). 1 mM IPTG (Promega, Madison, WI, USA, catalog #V3951) was then added to induce expression of the protein, and cells were grown for another 18 h at 37 °C. Cells were spun down and lysed using a detergent mix of triton X-100, sodium dodecyl sulfate, and sodium deoxycholate. Inclusion bodies containing RBD were dissolved in 3M urea (EMD Millipore, Burlington, MA, USA, catalog #1.08484.9029) and 3M guanidine hydrochloride (EMD Millipore, catalog #369079). RBD protein (22 kDa) was refolded and purified by buffer exchange using a tangential flow filtration system (EMD Millipore Pellicon 2 Mini Holder, catalog # XX42PMINI) with molecular weight cut-off membranes. Purity was determined by gel electrophoresis and endotoxin. Identity was confirmed by enzyme-linked immunosorbant assay (ELISA) using commercial anti-RBD antibodies (see details below).

### 2.2. Stabilizer Screening by Aggregation Inhibition

Denatured RBD was diluted to the target concentration of 100 µg/mL with or without additional stabilizers. 200 µL was plated to a polystyrene microplate (Sarstedt, Nümbrecht, Germany). Optical density at 320 nm (OD320) was measured with a SpectraMax M2e spectrophotometer (Molecular Devices) to measure back scatter, an indicator of aggregation, within 5 min. OD320 of RBD in sodium chloride (no stabilizer) was used as baseline as 100% aggregation. Aggregation inhibition percentage was calculated with the following equation: 100—(100 × (OD320 sample/OD320 no stabilizer)).

### 2.3. pH Profile and Buffer Screening by Dynamic Light Scattering

Denatured RBD was diluted to a target concentration of 100 µg/mL in appropriate pH and buffers. 1 mL of sample was added to the DLS cuvette and placed in the Zetasizer Nano ZS (Malvern, UK). The instrument was set to increase temperature in 5 °C increments and automatically take three DLS measurements after 5 min of equilibration at each temperature. Particle size data was then analyzed by Z-average and peak size by number and intensity.

### 2.4. Vaccine Formulation

Recombinant RBD bulk drug substance was added to aluminum adjuvants and mixed for 30 min at room temperature for adsorption. Commercial adjuvants from CRODA were used in this study. Alhydrogel^TM^ 2% and Alhydrogel^TM^ 2% 85 for aluminum hydroxide (AlOOH) and AdjuPhos^TM^ for aluminum phosphate (AlPO4). Mannan polysaccharide (Sigma, St. Louis, MI, USA, catalog # M3640) was then added to the suspension and mixed for 30 min. Methacrylic co-polymer (Eudragit L100-55, Evonik, Essen, Germany) was dissolved in saline and added to vaccine suspension for 1 h. The suspension was then slowly added to a solution of benzoic acid pH 4.0 to coacervate and coat the vaccine particles.

### 2.5. Dry Powder Vaccine Preparation

Liquid suspension vaccines were prepared with 2.5-fold higher concentrations of aluminum adjuvant and excipients to increase the solids amount and facilitate the drying process. For freeze-dried vaccines, a Labconco FreeZone Console Freeze Dryer (18 L) and a Labconco FreeZone Stoppering Tray Dryer were used. 0.7 mL of concentrated liquid suspension vaccine was added to the vials. The temperature was set to −40 °C and held for 30 min. The vacuum was then turned on and ran over night for ~16 h. The freeze dryer was then programmed to increase temperature 0.5 °C/min up to 20 °C.

For spray-drying, a Buchi B-290 mini spray dryer was used. Pressure was set to 150 psi, air flow to 40 mm with a rotary air valve, and heat at 100 °C. Once the system has reached steady conditions, the gear pump was set to 2 mL/min to pass liquid suspension vaccine through the hot column, and the nozzle sonicator was set to 9 to give a good conical-shaped spray. Powder was collected, weighed out, and transferred to vials.

### 2.6. Vaccine Analysis

#### 2.6.1. Adsorption

Protein and mannan adsorption were measured by spinning down the vaccine at 10,000 rcf for 5 min and testing the supernatant for protein and mannan. Total protein by BCA (ThermoFisher, Waltham, MA, USA, Catalog #23225 and #23235 for Micro BCA) is used to detect spike protein in the supernatant following the manufacturer’s instructions. The colorimetric anthrone assay is used for carbohydrate detection to test the presence of mannan in solution. Briefly, a 0.1% solution of anthrone is prepared in concentrated sulfuric acid and added to samples and standards in a microplate. The plate is cooled for 10 min at 4 °C and then heated on a heat block for 20 min at 100 °C. Absorbance at 620 nm is read with a spectrophotometer after letting the plate cool down for 20 min. Both protein and mannan assays were adapted to also measure adsorption of spike and mannan onto aluminum particles directly to confirm that total protein and mannan were recovered in the analysis.

#### 2.6.2. Particle Sizing by Laser Diffraction

Mastersizer 3000 was used to measure particle size (size range 10 nm to 3.5 mm). Between 0.5 mL and 1 mL of sample was added to the sample dispersion unit containing 40 mL of diluent. The instrument was set up to take 5 measurements by volume and by number while the sample is continuously circulating and mixing.

#### 2.6.3. Zeta Potential

Zetasizer NanoZS was used to measure zeta potential using laser Doppler electrophoresis, 3 measurements are measured for each sample.

#### 2.6.4. Sodium Dodecyl Sulfate Polyacrylamide Gel Electrophoresis (SDS-PAGE)

12% pre-cast gels from Sigma (TruPAGE, Catalog #PCG 2002) were used for RBD analysis (22 kDa). Vaccine samples were mixed with TruPAGE LDS loading dye buffer (Sigma, catalog # PCG 3009) and 1.25% β-mercaptoethanol to reduce the samples. The gel was run at 120V for 1.5 h, then stained with silver stain following the manufacturing kit protocol (Pierce^TM^, Appleton, WI, USA, catalog #24612). Image captured with ChemiDoc MP Imaging System (Biorad, Hercules, CA, USA).

#### 2.6.5. ELISA

High-binding 96-well microplates (Greiner Bio-One Microlon, Kremsmünster, Austria, catalog # 655061) were coated with an increasing amount of RBD in phosphate buffered saline (PBS) pH 7.4 and incubated at 37 °C for 1 h (same incubation conditions for all steps). After blocking with 2% BSA in PBS, rabbit anti-RBD polyclonal antibody (Cat# MBS1560390, Mybiosource) was added ×16,000 in BSA/PBS. Goat anti-rabbit IgG HRP (Abcam, Cambridge, CA, USA, cat#97240) was then added at ×40,000 and detected with TMB substrate. The microplate was read at OD450 after 30-min incubation in the dark, and the reaction stopped with 3 N sulfuric acid.

#### 2.6.6. Mice Studies

The handling and dosing of the animals were performed by Frontage Laboratories (Concord, OH, USA). For both studies, BALB/c mice (6 to 8 weeks old) were dosed with 0.4 mL of liquid suspension oral vaccine at Day 1 and Day 15 by oral gavage. Mice were sacrificed on Day 28, two weeks after the second dose. Animal study protocols were reviewed by the Frontage Laboratories ethical committee. IACUC approval dates for both studies were 23 July 2020, codes 038016 and 037904. Serum and intestinal lavage were collected by Frontage Laboratories, stored at −80 °C, and shipped to VaxForm for analysis.

#### 2.6.7. Intestinal Lavage Fluid (ILF) Collection

At termination, the small intestines were removed from each mouse, and a protease inhibitor cocktail solution (Sigma, catalog #P3840) was used to flush out the intestine contents (lavage). This was repeated with the same solution three times with 1 mL of solution. ILF was then transferred to a microcentrifuge tube stored at −80 °C until sent to VaxForm, Bethlehem, PA, USA.

#### 2.6.8. Antibody Analysis

RBD protein (1 µg/mL) was coated onto a 96-well microplate (Greiner Bio-One Microlon, catalog # 655061) for 1 h at 37 °C, washed 3 times, then blocked with 1% BSA (Pierce, catalog # 37525) in PBS for 1 h at 37 °C. Serum or ILF samples were added (dilutions ranging from 5-fold to 100-fold) and incubated for 2 h at 37 °C. Rabbit anti-mouse IgG-HRP (Sigma, catalog # A4416) or IgA HRP (Bethyl Laboratories, Montgomery, TX, USA, catalog # A90-103P) antibody was added to the plate for 1 h at 37 °C (×30,000 dilution for IgG-HRP and ×20,000 for IgA-HRP). After washing the plate one last time, bound antibodies are detected with a TMB substrate and incubated for 30 min in the dark at 25 °C. The reaction is stopped with 3 M sulfuric acid, and the plate is read at 450 nm. For titer calculation, a natural log regression curve was generated. Titer was calculated using a cutoff value of 0.3 for IgG (three times background OD450) and 0.2 for IgA. Titers were then normalized to the untreated group for each study to facilitate comparison. An unpaired *t*-test was performed to determine statistical significance between groups.

## 3. Results

### 3.1. RBD Bulk Drug Substance Stabilization

#### 3.1.1. Stabilizer Screening

**Figure 1:**
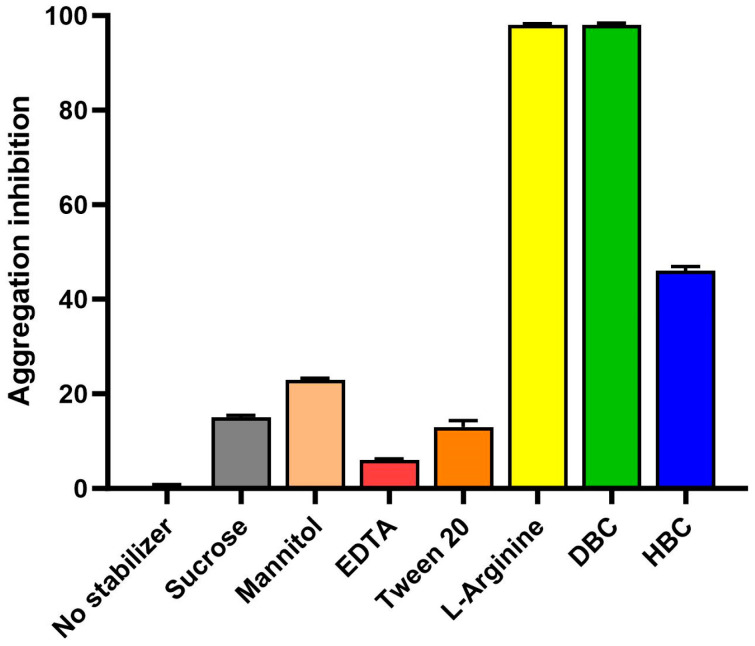
Stabilizer screening for RBD purification. RBD samples in 3 M urea, 3 M guanidine HCl were diluted to the 100 µg/mL target concentration in various stabilizer solutions. Sugars, sugar alcohols, and amino acids were tested at 200 mg/mL, 100 mg/mL EDTA, and 0.2% surfactants (Tween-20, Tween 80). Optical density at 320 nm (OD320) was measured as an indicator of protein aggregation. OD320 of RBD with no stabilizer was used as baseline for aggregation. Aggregation inhibition percentage was calculated, and the bar graph shows the average of triplicates with standard error bars.

The first step in developing this novel protein-based oral vaccine was to express and purify the recombinant RBD antigen. *Escherichia coli* (*E. coli*) was used as an expression host for the low-cost and scalable process. While *E. coli* is sometimes seen as an inferior expression host for not providing post-translational modifications, other studies have shown that recombinant RBDs from SARS-CoV expressed in mammalian cells, insects, or *E. coli* all elicited potent neutralizing antibodies and protective immunity [11]. The oral vaccine development work described in this paper was performed in 2020, when the only relevant literature available was from studies on SARS-CoV from the 2003 outbreak. The encouraging historical data on *E. coli*-expressed RBD in addition to the low-cost and scalable process justified the use of this host for this novel oral vaccine candidate. Inclusion bodies dissolution studies at VaxForm showed that a mix of 3 M urea and 3 M guanidine HCl was ideal to solubilize and denature the RBD. The protein of interest (22 kDa) was then purified using tangential flow filtration with molecular weight cut-off membranes. RBD protein was very unstable and formed large visible aggregates during the refolding process, as soon as the urea/guanidine HCl was removed. Excipient and stabilizer screenings were therefore conducted to establish buffer conditions that enhanced RBD stability and allowed proper refolding and purification. Back scatter analysis was used to screen stabilizers during the RBD refolding and purification process. Without added stabilizers, diluting the protein from its denatured form results in the formation of large aggregates that are detected by back scatter (320 nm). RBD samples in 3 M urea and 3 M guanidine HCl were diluted to the 100 µg/mL target concentration in various stabilizer solutions (all prepared in 20 mM Tris buffer pH 8.0 to avoid pH variability), or 150 mM NaCl only as a negative control (where maximum aggregation occurs). A total of 12 different stabilizers were screened from sugars, sugar alcohols, surfactants, and amino acids. Figure 1 shows a summary of the aggregation inhibition results (immediately following dilution) with a selection of stabilizers; sugars, sugar alcohols, and amino acids were tested at 200 mg/mL, 100 mg/mL EDTA, and 0.2% surfactants (Tween-20, Tween 80). Arginine and DBC (Dimethyl-β-Cyclodextrin) provided the most inhibition of aggregation (98%). L-Arginine was selected to move forward with in development. Further aggregation inhibition analysis was performed, monitoring RBD with L-arginine out to 24 h at room temperature with no observed aggregation.

#### 3.1.2. pH Profile and Buffer Screening

**Figure 2:**
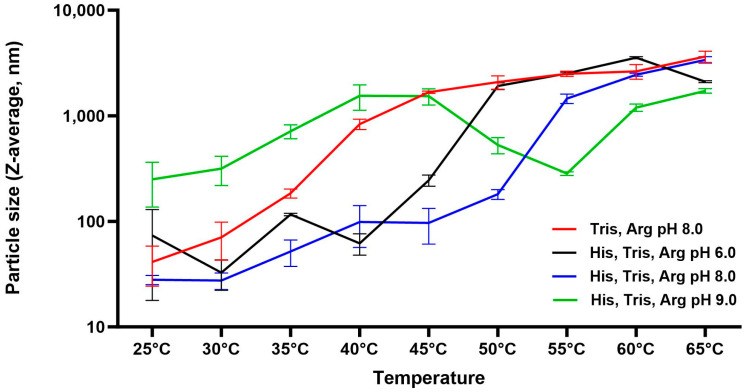
pH profile and thermal stability of RBD protein by particle size (Dynamic light scattering). RBD protein was formulated in 20 mM Histidine (His), 20 mM Tris, and 500 mM Arginine (Arg) buffers from pH 6.0 to 9.0. The hydrodynamic diameter of RBD was obtained by DLS (Zetasizer Nano ZS) at increasing temperatures from 25 °C to 65 °C. Averages of 3 measurements with standard error for selected conditions are shown.

Once arginine was determined as an appropriate stabilizer to stabilize the RBD protein, a pH profile and buffer screening were performed to determine the optimal bulk drug substance formulation. Dynamic light scattering (DLS) was used to detect sub-visible aggregation that cannot be detected by OD320 to complete the biophysical characterization of RBD. To conduct the pH profile, a combination of buffers that allowed pH adjusting from 6.0 to 9.0 was used so that only the pH would vary. RBD was diluted to 100 µg/mL in 20 mM Histidine, 20 mM Tris, and 500 mM Arginine buffer that was previously pH-adjusted to either pH 6.0, 6.5, 7.0, 7.5, 8.0, 8.5, or 9.0. Particle size at each pH was then monitored by DLS with Zetasizer NanoZS while increasing the temperature of the sample from 25 °C to 65 °C in 5 °C increments. The instrument allows detection of particles from 0.3 nm to 10 µm. The Z-average mean, also known as the cumulants mean or weighted intensity average, is the primary parameter produced by the DLS technique. The data is displayed as the hydrodynamic diameter of particles in nanometers (nm). Table 1 shows the Z-average of each pH at 25 °C before the temperature was increased. The Z-average of RBD in pH 9.0 was 250 nm, suggesting large aggregates and protein instability, while the Z-average of RBD at pH 8.0 was the lowest (28 nm), suggesting the most stable pH condition. To further investigate protein stability, aggregation was monitored with increasing temperature in Figure 2. This further demonstrated the optimal pH of 8.0 vs. 6.0 or 7.0. Additionally, it confirmed that histidine enhances stability, as shown by the red line; RBD formulated in Tris Arginine pH 8.0 increased much more rapidly in size versus the RBD formulated in Histidine Tris Arginine pH 8.0 (blue line). It was concluded that 20 mM Tris, 20 mM Histidine, 500 mM Arginine, pH 8.0 was the ideal buffer to purify and store the RBD bulk drug substance.

#### 3.1.3. Confirmation of Identity and Antigenicity

**Figure 3:**
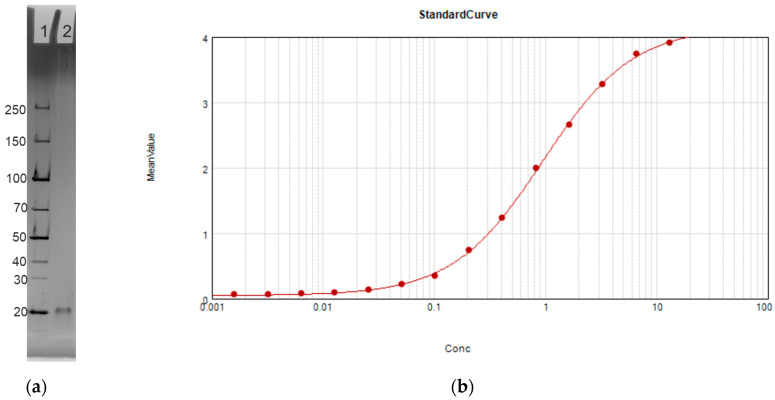
RBD characterization after purification. (**a**) 12% SDS-PAGE of RBD expressed and purified from *E. coli*. Lane 1: Protein ladder. Lane 2: RBD bulk drug substance. (**b**) ELISA standard curve obtained with RBD concentrations from 20 ng/mL to 10 µg/mL. X-axis shows concentration of RBD coated on the microplate in µg/mL, and Y-axis shows mean value by optical density OD 350.

SDS-PAGE and ELISA assays were developed to confirm that the expressed RBD antigen was functional. SDS-PAGE (Figure 3a) showed the recombinant purified RBD at the anticipated molecular weight of 22 kDa and free of potential contaminants from *E. coli*. ELISA (Figure 3b) confirmed antigenicity using commercially available RBD antibodies.

### 3.2. Oral Vaccine Formulation Development

#### 3.2.1. Adsorption to Aluminum Adjuvant

Previous studies in our laboratory have shown that both protein and mannan adsorption to aluminum (the carrier particle) are critical attributes of this oral vaccine platform. Mannans are polysaccharides and linear polymers of the sugar mannose derived from *Saccharomyces cerevisiae* known for their potential to elicit differential immune responses specific to antigens [12]. When adsorbed to aluminum adjuvant, mannan allows recognition by immune cells with CTL receptors and transcytosis through the intestinal lumen. In this study, antigen and mannan adsorption to aluminum hydroxide (AlOOH, Alhydrogel) and aluminum phosphate (AlPO4, AdjuPhos) was evaluated. Different ratios and order of addition were tested to ensure adsorption strength and capacity were well understood. Mannan and RBD mixed separately with aluminum oxyhydroxide and aluminum phosphate adjuvants, adsorbed 100% to each. However, when both RBD and mannan were mixed with aluminum adjuvant together, mannan adsorption to aluminum phosphate adjuvant was reduced to 62%. Further investigation revealed that the presence of arginine buffer caused lower adsorption for mannan to AlPO4 versus AlOOH. Another commercially available type of adjuvant, Alhydrogel 2% 85 (AlOOH—high binding), with higher adsorption capacity was also tested and resulted in the highest adsorption percentage (Table 2). Therefore, aluminum hydroxide 2% 85 was selected as an aluminum adjuvant/carrier particle for this vaccine.

#### 3.2.2. Coating Polymer Formulation

**Figure 4:**
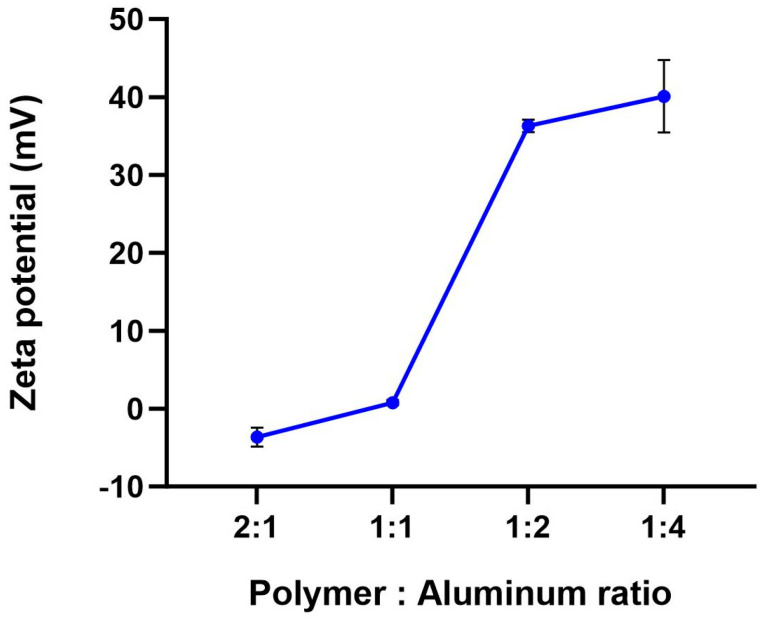
Effect of coating polymer to aluminum ratio on the microencapsulated oral vaccine surface charge. Vaccines were formulated with an increasing amount of coating polymer. Zeta potential was measured with Zetasizer Nano ZS using electrophoretic mobility.

**Figure 5:**
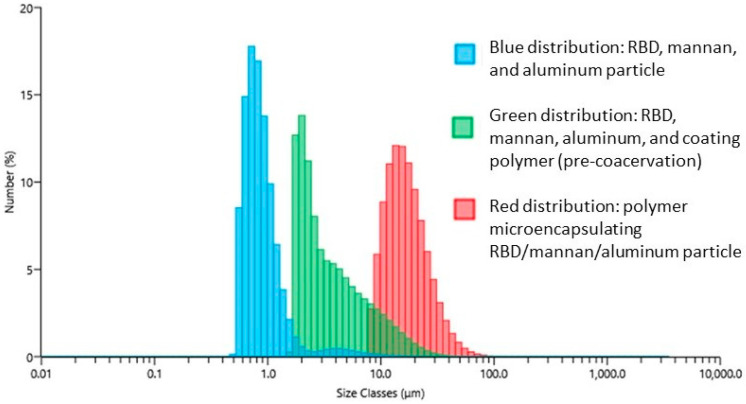
Particle size distribution overlay of formulations steps by laser diffraction. 0.5 mL of vaccine samples was added to the Mastersizer 3000 dispenser. Distribution graphs display the average of five measurements of particle size by number.

Once the antigen and mannan adsorption to aluminum was optimized, the addition of the coating polymer was tested to microencapsulate the vaccine and protect it from stomach degradation. Encapsulation of the vaccine particle (RBD, mannan, aluminum adjuvant) with enteric polymer is achieved by coacervation into low pH buffer. Various ratios of aluminum to polymer were evaluated to determine the optimal amount needed to encapsulate the vaccine without negatively impacting adsorption (both protein and mannan should remain adsorbent in the final formulation). The polymer used for this vaccine was methacrylate co-polymer Eudragit L100-55 (Evonik), an enteric polymer commonly used in pharmaceuticals that dissolves in the small intestines at pH > 5.5. Analytical methods utilized to evaluate proper encapsulation included pH, zeta potential, and particle size analysis by laser diffraction. To determine how much polymer was needed to encapsulate the vaccine particles, formulations were prepared with an increasing amount of coating polymer (0.05% to 0.4%, corresponding to 0.25:1, 0.5:1, 1:1, and 2:1 polymer to aluminum ratio) to a constant aluminum concentration. Figure 4 shows the effect of aluminum coating polymer ratio on surface charge/zeta potential (Zetasizer Nano ZS using electrophoretic mobility) following coacervation. A neutral surface charge is a marker of successful encapsulation. When too little coating polymer is added (0.5:1, 0.25:1 on the graph), the aluminum particles are not fully encapsulated, resulting in positively charged particles (Figure 4). pH measurements of these vaccines were also higher than 5.5, confirming a failed encapsulation process. These experiments led to selecting a 1:1 ratio of coating polymer to aluminum (1.0 mg/mL each). Particle size analysis by laser diffraction also confirms the proper ratio of polymers to aluminum as shown by the evenly distributed particles (red distribution, Figure 5).

#### 3.2.3. Final Drug Product Characterization

Once the formulation was determined, tests to simulate resistance of the vaccine to degradation by the conditions of the GI system were performed. The coating polymer needs to resist low pH so that the antigen and mannan are protected in the stomach. Once exposed to intestinal fluid (pH > 5.5), the enteric coating dissolves, and mannan can bind to microfold cells in the GALT to activate the mucosal immune response. Mannan and antigen need to remain adsorbed while exposed to intestinal fluid to efficiently target the immune cells. Therefore, in vitro experiments exposing the vaccine to simulated gastric fluid first and then simulated intestinal fluid were performed to ensure antigen and mannan remained adsorbed after dissolution of the coating polymer. The vaccine was mixed in 0.1 N HCl to simulate gastric fluid (SGF) for 1 h at 37 °C, then phosphate buffer at pH 6.8 for 1 h at 37 °C to simulate intestinal fluid (SIF). Zeta potential and adsorption were tested using a modified colorimetric BCA assay for the protein and anthrone assay for mannan. No protein or mannan were detected in the supernatant after the exposure to SGF, nor after exposure to SIF, suggesting proper encapsulation and polymer protection from the stomach environment. Zeta potential data (Table 3 below) also demonstrates protection of the vaccine particles in SGF (neutral charge) and dissolution in SIF (negative charge). 

### 3.3. Mannan Dose Response Immunogenicity in Mice

Once the final formulation process was determined, immunogenicity was evaluated in mice with a mannan dose response. Mice were administered 0.4 mL of the vaccine (10 µg RBD dose) by oral gavage and either no mannan/targeting molecule, 10 µg mannan, or 50 µg mannan twice, at Day 1 and Day 14. The antigen dose was selected based on the results of a small proof-of-concept study in BALB/c mice previously conducted. At Day 28, two weeks after the second dose, serum and intestinal lavage fluid were collected at termination. For titer calculation, a natural log regression curve was generated. Titers were calculated using a cutoff value of 0.3 for IgG (three times background OD450) and 0.2 for IgA. Titers were then normalized to the untreated group for each study (group 1) to facilitate comparison.

**Figure 6:**
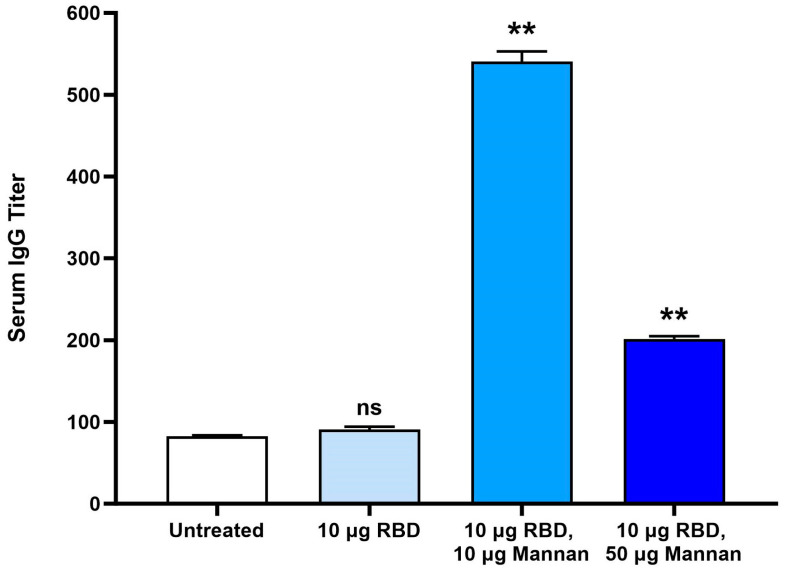
RBD-specific IgG serum titers in BALB/c mice. Mice (n = 10) were immunized on day 1 and 15 by oral gavage with either microencapsulated aluminum particles with either 10 µg RBD antigen only or microencapsulated aluminum particles with 10 µg RBD antigen and 10 µg or 50 µg mannan as GALT targeting molecule. Serum was collected on Day 28. Titers were measured by ELISA using in-house RBD for coating. For titer calculation, a natural log regression curve was generated with serum dilutions ranging from ×20 to ×540. ** represents *p* value < 0.01 by unpaired *t*-test analysis. 95% confidence interval vs. untreated. ns = not significant.

Figure 6 shows the titer levels of serum IgG and demonstrates the need for the mannan/GALT targeting molecule in the vaccine to induce an immune response. All treatment groups included aluminum particles and enteric coating. Vaccines either included no mannan, 10 µg mannan, or 50 µg mannan doses. The 10 µg RBD group (no mannan) did not induce antibody titers above the background set by non-vaccinated mice. 10 µg RBD, 10 µg mannan resulted in a 5-fold increase in IgG titers from baseline, while the 10 µg RBD, 50 µg mannan induced a close to 2-fold increase. Mucosal IgA analysis was not successful for this study as the collection of ILF did not result in usable samples. At the time, it was not known why more mannan resulted in lower IgG titers in the serum, but the later studies described below revealed a potential explanation.

### 3.4. Dry Powder Manufacturing

To further evaluate the feasibility of developing a highly stable COVID-19 oral vaccine to facilitate cost-effective distribution and storage, dry powder versions of the vaccine were produced and tested in vitro and in vivo. The liquid suspension vaccine was concentrated 2.5-fold to have enough solids to withstand the drying process. The addition of sugars such as sucrose and cyclodextrins was tested during the optimization of the drying process but was not found to enhance powder stability. Particle size, pH, and reconstitution time were measured before and after the drying process to ensure the process did not negatively impact the vaccine particles. Table 4 below also displays assay results before and after the drying process, suggesting vaccines maintained their enteric coating during the drying process. Both freeze-dried and spray-dried powders were easily reconstituted in water.

### 3.5. Immune Response Profiles of Liquid vs. Dry Powder Vaccines in Mice

Vials from each powder presentation and liquid suspension vials were shipped to Frontage Laboratories. The day of administration, the technicians reconstituted each vial with the proper diluent as per our protocol to obtain the right dosage, and mice were orally gavaged with the resulting vaccine suspension.

**Figure 7:**
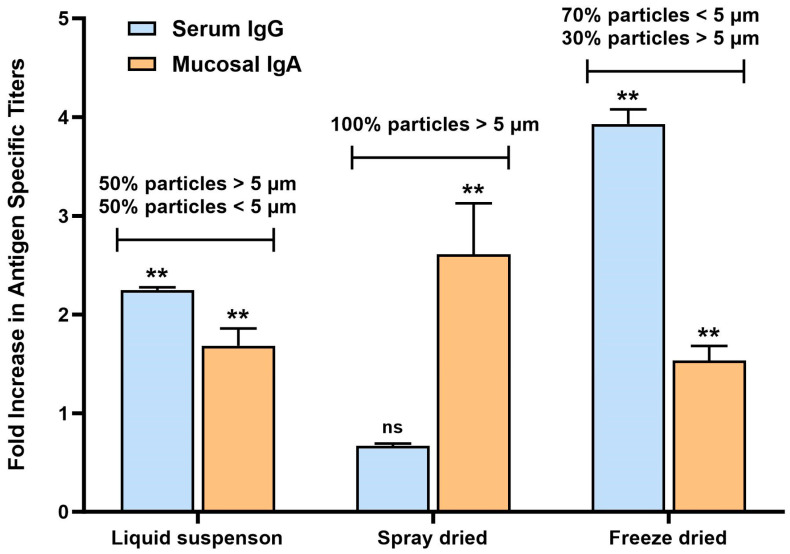
RBD-specific serum IgG and mucosal IgA titers in BALB/c mice after 2 doses of oral liquid suspension vaccine. Mice (n = 10) were immunized on days 1 and 15 by oral gavage with 10 µg RBD and 10 µg mannan. Serum and intestinal lavage were collected on day 28. ELISAs were performed to detect RBD-specific antibody levels. Titers were normalized to the untreated group as a fold increase. ** represents *p* value < 0.01 by unpaired *t*-test analysis, 95% confidence interval. ns means not significant.

Serum IgG and mucosal IgA (from intestinal lavage samples) titers (normalized to the untreated group) are shown in Figure 7 for all 3 types of vaccines (not force-degraded). The RBD-specific antibody titer profile of each vaccine version revealed interesting differences. Liquid suspension vaccine resulted in a balanced serum IgG/mucosal IgA response, while spray-dried vaccine induced a strong mucosal response/no serum IgG and freeze-dried vaccine serum IgG-biased response. When analyzing the particle size by laser diffraction of the vaccines after exposure to SIF, a correlation was found between particle size and immune response profile, as shown in Figure 6. The liquid suspension vaccine particle size distribution had 50% of particles below 5 µm and 50% above 5 µm. The spray-dried vaccines contained particles all larger than 5 µm, and the freeze-dried vaccine contained mostly smaller particles (70% below 5 µm). Other studies that investigated vaccine release in the GALT to stimulate mucosal IgA antibody response found that microspheres less than 5 µm in diameter tend to result in systemic response due to their propensity to disseminate to systemic lymphoid tissues, whereas vaccine microspheres larger than 5 µm induced a predominant mucosal IgA response as they remain in the intestinal mucosa for longer periods of time [13,14]. Results in this mouse study confirm this observation and reveal that particle size in SIF is a key critical attribute for the vaccine and should be monitored more closely to obtain the intended type of immune response.

### 3.6. Thermostability

#### 3.6.1. Real Time In Vitro Stability

**Figure 8:**
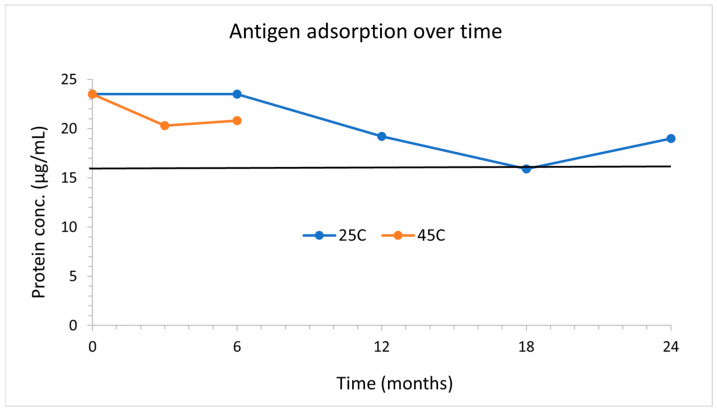
RBD adsorption over 2 years at 25 °C and 6 months at 45 °C. Vaccines are exposed to simulated intestinal fluid, spun down, and a total protein assay is performed both on the supernatant and aluminum to determine protein adsorption.

The vaccine stability was monitored at 25 °C for 2 years and 45 °C for 6 months by the protein and mannan adsorption after exposure to simulated intestinal fluid. Figure 8 below shows adsorption of RBD remained above 80% (16 µg/mL) for 2 years at 25 °C and 6 months at 45 °C.

#### 3.6.2. Accelerated Stability: In Vitro Study

An immunogenicity study in mice was conducted to evaluate and compare the performance of liquid suspension vaccines versus freeze-dried and spray-dried vaccines and their resistance to heat-forced degradation. After some initial in vitro testing that showed very strong resistance to heat, it was decided to expose the vaccines at 60 °C for 14 days to obtain a good amount of degradation and thermostability of the 3 types of vaccines. Two sets of vaccines were prepared and either stored at 25 °C or 60 °C for 14 days. After the 14 days, all vials were stored back at 25 °C and shipped within a week to Frontage Laboratories for dosing. Vaccines were prepared as described earlier, with a target formulation at dosing of 25 µg/mL RBD, 25 µg/mL mannan with 1 mg/mL aluminum adjuvant, and 1 mg/mL coating polymer. This is the equivalent of a 10 µg RBD, 10 µg mannan dose (0.4 mL, administered twice), since the first animal study suggested this formulation resulted in the best antibody titer response. The liquid suspension formulation was prepared 2.5-fold concentrated to have enough solids to withstand the drying process. Those vaccines were then filled in glass vials as liquid suspension for the freeze-drying process. For the spray-drying process, a liquid suspension bulk was spray-dried (following procedure described above), collected as powder, then filled in vials by weight. The correct weight was determined by reconstituting some of the powder in water and evaluating the protein and mannan content on the carrier particle. Half of the vials prepared for each vaccine presentation were then exposed to 60 °C for 14 days. Data had shown that the liquid suspension vaccine was stable at 45 °C for several months. It was anticipated that the powders would be even more stable; therefore, 2 weeks of heat-force degradation at high temperature was chosen to obtain degraded material that would result in differences in vivo. The vaccines were also characterized before and after forced degradation by particle size, zeta potential, pH, and adsorption stability (Table 5 below).

The exposure to high temperatures did not impact vaccine appearance, reconstitution time, pH, mannan adsorption, or zeta potential significantly. Vaccines exposed to 60 °C showed a trend of increase in protein adsorbed onto aluminum particles. This observation can be explained by protein degradation and structural changes detected by the BCA assay used for this analysis. An animal study was conducted next to evaluate whether the changes observed in the in vitro assays correlated with lower immunogenicity.

#### 3.6.3. Accelerated Stability: In Vivo Study

**Figure 9:**
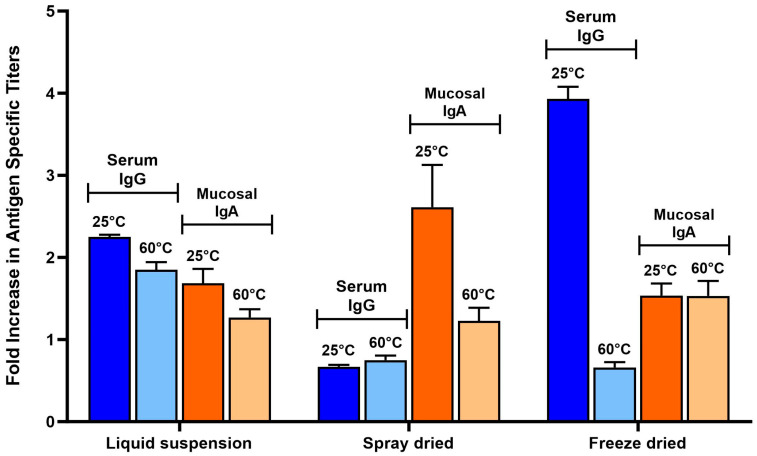
Heat force degradation immunogenicity study of liquid suspension versus spray dried and freeze-dried powder vaccines. RBD-specific serum IgG and mucosal (intestinal lavage) IgA titers were analyzed from mice (n = 10) that were immunized on days 1 and 15 by oral gavage with either liquid suspension vaccine, spray-dried vaccines reconstituted in water, or freeze-dried vaccines reconstituted in water after two weeks of storage at either 25 °C or 60 °C. Serum and intestinal lavage fluid (ILF) were collected on Day 28. ELISAs were performed to detect RBD-specific antibody levels, serum IgG, and mucosal IgA (ILF). Titers were normalized to the untreated group as a fold increase.

Figure 9 below shows the antibody titers induced by the vaccines before and after being exposed to the heat-forced degradation conditions. Titers of vaccines at 25 °C in Figure 9 and Figure 7 are the same. Bars were added in this Figure again to facilitate direct comparison of normal (25 °C) vs. heat-forced degraded vaccines (60 °C). Results suggest that the heat impacted the dry powder vaccines more than the liquid suspension vaccine. Spray-dried vaccines exposed to 60 °C resulted in a decrease in mucosal IgA from 2.5-fold for the 25 °C vaccines to 1.2-fold. The serum IgG response of freeze-dried vaccines was abolished by the exposure to 60 °C (compared with a 4-fold increase of 25 °C vaccines) but did not impact the mucosal IgA response (both 25 °C and 60 °C stored vaccines resulted in significant levels of IgA, *p* value < 0.01). The impact of heat exposure on the immunogenicity of the liquid suspension vaccine was not as significant. Mucosal IgA, while trending lower, was not significantly lower (*p* value 0.06). Serum IgG levels of 60 °C exposed vaccine were lower than 25 °C (*p* value < 0.015) but were still higher than baseline/untreated mice (*p* value 0.0004).

## 4. Discussion

Next-generation vaccines are needed to provide better protection from respiratory viruses such as SARS-CoV-2 as well as improve global access, especially during outbreaks and pandemics [15,16]. Oral vaccination offers significant advantages for distributing and administering the vaccine as well as enhancing protection by providing immunity at mucosal surfaces, the site of infection. Our study demonstrates the feasibility of using a C-type lectin receptor agonist in an oral subunit vaccine to induce mucosal and systemic responses. To our knowledge, this is the first time this has been performed. The immunogenicity studies also confirmed the observation made by other vaccine researchers that oral vaccine particle size can impact mucosal versus systemic immune responses. Particles less than 5 µm in diameter tend to result in systemic responses due to their propensity to disseminate to systemic lymphoid tissues, whereas vaccine particles larger than 5 µm induce a predominant mucosal IgA response as they remain in the intestinal mucosa for longer periods of time [13,14]. In this study, the spray-dried vaccines (formulation with particles larger than 5 µm) demonstrated the platform’s potential in inducing strong mucosal IgA responses. Mucosal immunity has been shown to play a key role in providing control of viruses in the upper respiratory tract and preventing infection [17,18]. Several studies have shown that secretory polymeric IgAs have superior neutralizing activity compared with serum IgG and are correlated with enhanced protection from infection [19]. Results from this study further demonstrate the importance of robust vaccine formulation development and characterization to fully understand the critical attributes and their impact on immunogenicity. Future studies will focus on leveraging the particle size attribute during formulation development to induce strong mucosal immune responses and evaluating its impact on protective immunity. Overall, more dedicated studies are needed to further understand the role of mucosal immunity during infections and vaccination and establish mucosal correlates of protection to facilitate the development of mucosal vaccines.

Limitations to this study include the use of an animal model that does not fully reflect the human GI tract. Animal studies with a platform that is pH-dependent for release are very challenging since most rodents have unique GI systems. However, a Phase I human clinical trial was conducted with this vaccine candidate and demonstrated excellent safety profile and good immunogenicity (manuscript in preparation). Neutralizing antibodies and cell-mediated immunity were not measured in this study due to sample limitations. While oral secretory IgA was not evaluated in this study, other studies have shown that plasma cells in the gut can disseminate to other mucosal surfaces, such as the oral cavity [20]. VaxForm’s Phase I clinical trial demonstrated successful oral IgA induction using the same delivery platform. Future studies will focus on developing an oral COVID-19 booster vaccine (booster to injected mRNA vaccines) to improve protection and reduce transmission using the full spike protein, where additional mucosal and cellular response analyses will be performed.

This study shows that VaxForm’s oral vaccine delivery platform is compatible with an oral COVID-19 vaccine using RBD as an antigen, mannan as the targeting molecule, and methacrylate co-polymer Eudragit L100-55 as the enteric polymer. All active and non-active components of the vaccines are used in other pharmaceuticals and safe for use in humans. Mannans are widely used in the food industry—their nontoxicity allows usage in the pharmaceutical, biomedical, and cosmetic industries [21]. While there are no oral vaccines with aluminum adjuvant currently approved by the FDA, aluminum is found in other approved drugs. Aluminum hydroxide is often administered orally for the temporary relief of heartburn or gastroesophageal reflux and to treat oral mucositis in the form of mouthwash. Aluminum hydroxide, when used as an antacid, can be taken orally 5 to 6 times daily, not to exceed 3.84 g per 24 h [22]. Our vaccine formulations for human use would contain at most 10 mg of aluminum per dose, much below the daily intake limit. Currently, all oral vaccines approved for licensure or in clinical development use inactivated, live-attenuated, or vector-based technologies. The advantage of VaxForm’s platform includes using a well-established scalable manufacturing process (recombinant protein) and ingredients with good safety records, removing the greatest current barriers to late-stage oral vaccine development: large-scale manufacturing and regulatory approval.

This study also demonstrates the feasibility of developing a vaccine resistant to high temperatures. In vitro assays demonstrated 2-year stability at 25 °C and 6 months at 45 °C. In vivo evaluation in mice under force-degradation conditions of liquid suspension vaccines and dry powder vaccines showed differential impacts on physical stability as well as the type of immune response. The liquid suspension vaccine overall seemed to have the highest resistance to the high temperature exposure. However, it is possible that the spray-drying, and freeze-drying processes were not fully optimized and could be improved to result in equivalent, if not higher, stability than the liquid suspension. Regardless of vaccine presentation, removal of the cold chain for distribution and storage would be a game changer for vaccines globally. Additionally, the ease of administration of oral vaccines removes the need for medical professionals and mass vaccination clinics. These advantages are greatly needed when attempting to increase population compliance for annual booster vaccines such as influenza and COVID-19.

## 5. Conclusions

This study demonstrated that VaxForm’s oral vaccine delivery platform can successfully induce mucosal and systemic immune responses in mice and proved the feasibility of developing a shelf-stable, orally administered protein-based COVID-19 vaccine. Results from this study highlight the importance of formulation development and analysis by showing the impact of the vaccine particle size on the immune response profile (systemic-biased versus mucosal-biased). In addition to reducing infection rates and transmission of the virus, this oral vaccine has the potential to greatly improve global access to vaccines by eliminating cold-chain requirements and enhancing compliance by allowing self-administration and reducing systemic side effects.

## Figures and Tables

**Table 1:** RBD particle size (Z-average) at various pHs. RBD stock was diluted to 100 µg/mL in 20 mM Histidine and 20 mM Tris, pH 6.0 to 9.0. particle size was measured with Zetasizer Nano ZS by Dynamic Laser Scattering. Z-average is the weighed intensity.

pH	Z-Average (nm)
6.0	73.7
7.0	77.3
8.0	28.0
9.0	250.4

**Table 2:** Protein and mannan adsorption to aluminum adjuvants.

Aluminum Adjuvant	% Protein Adsorbed	% Mannan Adsorbed
AlOOH high binding	95.3	93.7
AlOOH	95.7	78.7
AlPO4	78.7	62.2

**Table 3:** Zeta potential of vaccine in formulation after exposure to simulated gastric fluid (SGF) and exposure to simulated intestinal fluid (SIF).

	Zeta Potential (mV)
Vaccine formulation	−4.5
Vaccine in SGF	6.4
Vaccine in SIF	−29.9

**Table 4:** Vaccine characterization before and after spray-drying and freeze-drying processes.

	Before Drying (Liquid Suspension)	Spray-Drying	Freeze-Drying
pH	4.1	4.4	4.3
Protein and mannan adsorption	>80%	>80%	>80%
Particle size in SIF	80% of particles between 1 µm and 10 µm	80% of particles between 1 µm and 10 µm	80% of particles between 1 µm and 10 µm
Reconstitution time	N/A	<3 min	<3 min

**Table 5:** Characterization of liquid suspension, spray dried, and freeze-dried vaccines before and after heat-forced degradation.

Vaccine	Reconstitution Time	pH	Adsorbed Protein (µg/mL)	Adsorbed Mannan (µg/mL)	Zeta Potential
Liquid suspension 25 °C	<1 min	4.1	27.8	25.0	−5.0 mV
Liquid suspension 60 °C	<1 min	4.2	39.4	23.7	−9.0 mV
Spray-dried 25 °C	<1 min	4.4	32.1	18.9	−6.4 mV
Spray-dried 60 °C	<1 min	4.5	51.1	25.0	−2.3 mV
Freeze-dried 25 °C	<1 min	4.3	28.1	23.0	−8.9 mV
Freeze-dried 60 °C	<1 min	4.4	38.6	22.4	−7.6 mV

## Data Availability

The data presented in the article can be available on request from the corresponding author to any researcher wishing to use them for non-commercial purposes and will have to be approved by VaxForm legal prior to sharing. The data are not publicly available due to the proprietary nature of the work.
